# Ischemic stroke is an instigator of cardiac dysfunction: the interleukin-1β pathway

**DOI:** 10.1038/s41392-024-02040-7

**Published:** 2024-11-26

**Authors:** Renate Schoenenberger-Berzins, Andreas Janis Schoenenberger, Franz H. Messerli

**Affiliations:** Department of Cardiology, Luzerner Kantonsspital, Luzern, Switzerland

**Keywords:** Cardiology, Innate immune cells

In a recent study published in *Cell*,^1^ Simats et al. showed in a comprehensive series of elegant, mostly experimental studies, that stroke triggers epigenetic changes in innate immune cells which thereafter can affect the heart.

The devastating impact of ischemic stroke is known to go far beyond the initial brain injury, with much of its burden arising from comorbidities that develop subsequently. Cardiac complications are exceedingly common in the first few days after an ischemic stroke.^2^ Although there may be a common trigger and a mechanism, yet not fully understood, Simats et al. document that there is participation of innate immune cells.^1^

**Fig. 1:**
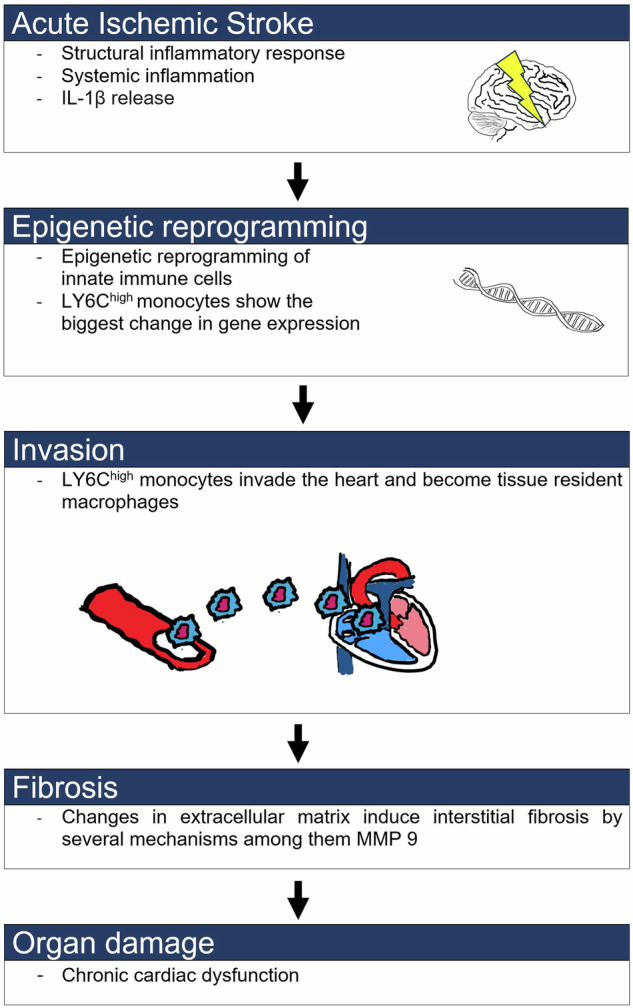
Illustration of the Interleukin-1β pathway

In their study, Simats et al. identify myeloid innate immune memory as a key factor contributing to dysfunction in distant organs following stroke (Fig. 1). Several weeks after the brain lesion monocytes/macrophages in many organs still show pro-inflammatory changes. Specifically, LY6C^high^ monocytes showed the largest change in gene expression, which may have led to an increase of tissue-resident macrophages in the heart. Change in gene expression of LY6C^high^ monocytes is known to be associated with higher MMP9 activity, which is responsible for extracellular matrix (ECM) remodeling and subsequently inducing fibrosis.^3^ In both mice and stroke patients Simats et al.,^1^ showed these changes to be associated with cardiac fibrosis and dysfunction. IL-1β was identified as a major driver of epigenetic alterations in innate immune memory. Eventually the authors documented that these changes could be transferred to naive mice. Either transplantation of bone marrow (BM) from stroke mice or exposition to high levels of IL-1β was leading to similar cardiac dysfunction.

Importantly, by neutralizing IL-1β after stroke or blocking pro-inflammatory monocyte migration using a C-C chemokine receptor type 2 and 5 antagonist, post-stroke cardiac dysfunction could be prevented. Simats et al.^1^ suggest that these immune-targeted therapies could offer a novel approach for preventing various IL-1β-mediated comorbidities, providing a framework for secondary prevention through immunotherapy.

The study by Simats et al.^1^ strongly supports the growing concept that systemic inflammation caused by acute events can result in persistent inflammatory memory. This memory can, in turn, trigger additional inflammatory processes that contribute to the development of comorbid diseases.^4^ Identifying common pathways that initiate maladaptive trained immunity may hopefully lead to specific strategies for treating or reducing inflammatory comorbidities.^4^

As convincing as this chain of experiments documenting the cardiac after-effects of an ischemic stroke are, there are a few points to be scrutinized:

1. Could non-stroke brain injuries (e.g., trauma, surgery, infection) trigger similar events?
  The *Cell* paper documents that giving IL-1β antibodies after stroke to mice, partially reduces changes in gene expression of innate immune cells. However, IL-1β is a cytokine involved in many signaling processes of the immune system and inflammatory responses; after myocardial infarction LY6C^high^ monocyte orchestrate both inflammatory and reparative processes in the heart. However, Simats et al.‘s study focused mainly on ischemic stroke, leaving the above question unanswered.^1^ They nevertheless showed, that other cytokines such as Il-12 and Interferons may be involved.^1^
2. Is post-stroke organ dysfunction specific for the heart or are there other organs affected?
  Changes in gene expression of LY6C^high^ monocytes may be associated with tissue residency of macrophages. Simats et al.,^1^ found the invasion of circulating LY6C^high^ monocytes to the heart and changes in gene expression of those monocytes, related to tissue residency. The conclusion formed by the authors reads: “Taken together, these results suggest that stroke chronically promotes the recruitment of circulating LY6C^high^ monocytes to the heart, which might further differentiate into tissue-resident macrophages”. Of note however, Simats et al. did find changes in monocytes/macrophages population of other organs than the heart, being very distinct e.g. in the liver.^1^ However the invasion of LY6C^high^ monocytes seemed to be specific for the heart.^1^
3. Could colchicine, an IL-1, IL-6, and IL-18 inhibitor,^5^ serve as non-specific cardioprotective therapy?
  According to Simats et al. treating patients with IL-1β antibodies may increase the risk of infections.^1^ In an attempt to bypass this risk they gave cenicriviroc (cvc) which is known to prohibit the invasion of circulating myeloid cells into various organs. They showed that daily treatment with cvc reduced recruitment of monocytes to heart. However, the question remains, whether these effects could to some extent, be mitigated by giving e.g. canakinumab, an IL-1β antibody, or a less specific drug like colchicine. Several recent trials, such as CANTOS (Canakinumab Anti-Inflammatory Thrombosis Outcomes Study, COLCOT (Colchicine Cardiovascular Outcomes Trial) and LoDoCo2 (Low Dose Colchicine 2), have indeed shown that suppressing inflammation can improve outcomes in ASCVD.
4. Several questions remain:
  Is this the principal pathogenetic mechanism causing what is currently known as the stroke-heart syndrome?
  Is stroke-heart syndrome a true finding or simply the result of the fact that echocardiography is mandatory in stroke work-up?
  Is there potentially a bidirectional communication in that the same mediators could elicit via a heart-to-brain axis, a heart-brain syndrome?
  Should we now launch clinical trials comparing canakinumab or colchicine against placebo for prevention of the stroke-heart syndrome?

In conclusion, the Cell paper showed step-by-step that ischemic stroke causes an inflammatory memory, triggering monocytes to infiltrate the heart and to cause cardiac dysfunction. In elucidating this pathway, Simats et al. have provided us with a fascinating novel pathogenetic and therapeutic hypothesis.^1^

To us, it fulfils the classic criteria of an Emmenthal cheese hypothesis: It looks good, it smells good, it tastes good, but still there are large holes! However, we are exceedingly confident that these, hole by hole, will be taken care of by further clinical and experimental research efforts and that what we would like to identify as the Interleukin-1β Pathway will become firmly established in Cardiovascular Medicine.
